# A CRISPR/Cas9 system adapted for gene editing in marine algae

**DOI:** 10.1038/srep24951

**Published:** 2016-04-25

**Authors:** Marianne Nymark, Amit Kumar Sharma, Torfinn Sparstad, Atle M. Bones, Per Winge

**Affiliations:** 1 Department of Biology, Norwegian University of Science and Technology, N-7491 Trondheim, Norway

## Abstract

Here we report that the CRISPR/Cas9 technology can be used to efficiently generate stable targeted gene mutations in microalgae, using the marine diatom *Phaeodactylum tricornutum* as a model species. Our vector design opens for rapid and easy adaption of the construct to the target chosen. To screen for CRISPR/Cas9 mutants we employed high resolution melting based PCR assays, mutants were confirmed by sequencing and further validated by functional analyses.

Marine microalgae are responsible for 40% of global primary production and capture more CO_2_ than all the rain forests combined^1^. Diatoms (Bacillariophycea) are the most important group of eukaryotic microalgae, and have enormous ecological significance^2^,^3^,^4^. This group of microalgae can also be used as a source of lipids for biofuels, as well as other applications in bio-, nano- and environmental technology^5^,^6^. Studies of microalgae have been hampered by inefficient genome editing methods restricting functional analyses. An improved system for generation of mutants is important for 1) functional analyses of genes/proteins to improve our understanding of diatom biology and 2) for strain improvement for biotechnological applications. In previous years it has only been possible to create non-targeted knock-down (RNAi) or overexpression lines in diatoms, but recently two independent groups demonstrated for the first time precise targeted genome editing of the *Phaeodactylum tricornutum* genome^7^,^8^. The modifications of the diatom genome were achieved using protein-based systems involving meganucleases and transcription activator-like effector nucleases (TALENs) that have specific DNA binding activities. These systems are labour intensive and costly, so we set out to establish the much simpler and inexpensive CRISPR/Cas9 technology for genetic engineering of diatom genomes^9^.

We developed a highly efficient CRISPR/Cas9 based system optimized for creating stable targeted gene knockouts in the marine diatom *P. tricornutum.* The two components necessary for this system to function, the Cas9 nuclease and a guide RNA directing the nuclease to a specific DNA sequence, were expressed from the same vector. The diatom codon-optimized *Cas9* was driven by a promoter for a fucoxanthin chlorophyll a/c-binding protein gene (*LHCF2*), and the single guide RNA (sgRNA) was under control of the *P. tricornutum* U6 promoter. The *Cas9* and the sgRNA modules were cloned into the same vector (pKS diaCas9_sgRNA; Supplementary Fig. 1) to increase the probability of co-delivery during biolistic transformation of the *P. tricornutum* cells. By exploiting two BsaI cutting sites located within the sgRNA cassette (Supplementary Fig. 1), a small adapter can easily be inserted into the sgRNA, making it target-specific to the gene that it is desired to disrupt.

As a proof of concept we targeted the chloroplast signal recognition particle 54 (CpSRP54; Draft ID Phatr2_35185), a member of the chloroplast signal recognition particle (CpSRP) pathway, for CRISPR/Cas9-based disruption. By using a combination of high resolution melting (HRM) analyses and Sanger sequencing we detected a variety of indels at the target site (located in the conserved SRP54 N-terminal domain (Fig. 1A)) in 8 out of 26 transformants (Fig. 1B,C), giving a mutation frequency of 31%. In addition to the CRISPR/Cas9-mediated mutagenesis of the *CpSRP54* gene, we have successfully knocked out two other genes, using two different sgRNAs (targets) per gene. The mutation frequencies in these experiments were also high, ranging between 25–63% (unpublished data). All transformants cannot be expected to contain mutations because: 1) The non-selectable pKS diaCas9_sgRNA plasmid is co-transformed with the pAF6 plasmid to confer resistance to zeocin^10^. According to literature 30–40% of the transformants are expected to contain only the pAF6 plasmid^10^, and no targeted gene mutations will occur. 2) Biolistic-mediated transformation is known to cause fragmentation and incomplete incorporation of the Cas9 gene^11^. Intact Cas9 and sgRNA is a necessity for CRISPR mutagenesis to take place. In plants, the incomplete incorporation of Cas9 has been suggested as a likely explanation for the much lower mutation efficiencies (5–12%) reported when using biolistic bombardment for introduction of the Cas9 and sgRNA^11^,^12^,^13^, compared to *Agrobacterium*-mediated transformation (20–90%)^14^,^15^,^16^,^17^.

The detected indel mutations in the *CpSRP54* gene included single nucleotide (nt) insertions, a variety of mostly short deletions and in one case a 212 bp insertion (Fig. 1C). As reported for large insertions generated by repair of TALEN induced double strand breaks^7^, the 212 bp insertion found here also consisted of fragments of the vectors used for transformation. HRM and sequencing data indicate that in colonies where indels are detected, cells with mutations completely dominate over cells with wild type (WT) alleles (Fig. 1B,C). Amplified genomic DNA from colonies with targeted mutations was TOPO-cloned and 7–8 clones from each colony were sequenced. Only three out of a total of 62 clones contained the WT allele.

Chromatograms produced from direct sequencing of PCR product confirming insertions in colony M1, M2 and M8 showed clear peaks with low background levels also after the site of the insertion (Fig. 1B). The 21 bp deletion detected in *CpSRP54* from colony M5 could be identified by direct sequencing of the PCR product (Fig. 1B). The data imply that the vast majority of the cells in these colonies are mutants having identical biallelic mutations, suggesting a double-strand break (DSB) induced gene conversion mechanism^18^. Sequencing of the cloned PCR products from M1, M2, M5 and M8 detected only one type of mutation in each colony, supporting this assumption (Fig. 1C). The results for the M1, M2, M5 and M8 mutants suggest that whereas the DSB in one allele is repaired by the error-prone Non-homologous end joining (NHJ) pathway, the DSB in the other allele might be repaired by homologues recombination (HR) using the newly mutated allele as template for repair. The limited number of different indels in cells derived from individual transformants suggests that the DSBs and genome editing start already within the initially transformed cell. When several different mutated alleles are found within a colony (Fig. 1C), implying that a few cell divisions have occurred before the first mutagenic event, the high efficiency of the CRISPR/Cas9 system still causes an almost complete elimination of the WT sequence. In contrast, the percentage of mutant *P. tricornutum* cells within colonies shown to have targeted gene modifications induced by meganucleases was only 1–15%^7^. For TALEN the equivalent number was 6–80%^7^.

Proteins of the CpSRP pathway are involved in assembly and integration of proteins into the thylakoid membrane^19^. To the best of our knowledge, no CpSRP54 knockout mutant has previously been reported in microalgae, but based on information from higher plants we expected such a mutant to have an easily recognizable phenotype connected to alterations of the photosynthetic apparatus^20^,^21^. Sensitivity to high light has also been reported in CpSRP54 mutants in higher plants^21^. Since high light sensitivity would be an easy way to show a functional effect of the disruption of the *CpSRP54* gene in *P. tricornutum,* we decided to provoke light-induced damage to photosystem II (PSII) by exposing mutants to high intensity blue light (BHL). The photosynthetic efficiency (measured as the maximum quantum yield, F_v_/F_m_) was monitored during the period of BHL stress, and through the following recovery period in dim white light (WL) conditions (Fig. 2). Exposure to BHL for 1 h caused a greater decrease in F_v_/F_m_ in the CpSRP54 mutant cultures than in WT cultures. Both mutants and WT cells showed an almost complete recovery after 3 h of dim WL conditions (Fig. 2). The increased sensitivity to high intensity light in mutant cultures is in support of the HRM and sequencing data indicating that cells with mutations completely dominate over wild type within the culture.

“Clean” CpSRP54 mutant lines originating from single cells with biallelic mutations have also been isolated by spreading out highly diluted cell suspensions from the original cultures on agar plates, and repeating the screening process on the resulting colonies. These cultures will be used for future characterization of the CpSRP54 mutants. Gene expression analyses detected high levels of sgRNA in all of the clean CpSRP54 mutant lines, and similar high levels of *Cas9* mRNA in all but one of the same cultures (Supplementary Table 1). The transcript levels of *Cas9* and sgRNA were within the same range as the endogenous Light Harvesting Complex gene *LHCX1* (XM_002179724) (Supplementary Table 1), known to be one of the most abundant mRNAs in *P. tricornutum*^22^. The transcriptional analyses confirm high gene expression and stable integration of the *Cas9* and sgRNA genes into the *P. tricornutum* genome. Constitutively expressed Cas9 protein has been reported to be toxic for *Chlamydomonas reinhardtii*^23^. Clean *P. tricornutum* CpSRP54 mutant lines grow at a similar or slightly slower rate (Div. per day: ~1.5–2.0 day^−1^; Supplementary Table 2) than WT (~2.0 day^−1^) in dim WL conditions. Transcriptional analyses of the clean mutant culture M8.1, originating from M8, showed that the sgRNA, but not the *Cas9* gene is expressed in this culture. Still, the M8.1 culture has the lowest growth rate of all the mutant lines (~1.5 day^−1^). The above described results indicate that the observed growth differences are not likely to be connected to a toxic effect of constitutive Cas9 expression.

Diatoms are important as the major group of primary producers of the oceans and as a promising and basically unexploited group of eukaryotic organisms for bio-production and biotechnological exploitation. We have developed a very efficient genome editing and selection method for the diatom *P. tricornutum*, which can be potentially adapted for use in other microalgae. We anticipate our method to be useful for all kinds of functional studies in microalgae, and also for engineering of specific traits like modified light-harvesting antennas and lipid profile.

## Methods

### Growth conditions

Axenic *P. tricornutum* cells (CCMP2561) were grown as described previously^24^ in a controlled *in vitro* growth room under a 16 h photoperiod at 22 °C, 65 μmol photon m^−2^ s^−1^.

### Vector construction

A codon-optimized Cas9 protein (diaCas9) modelled after hSpCas9^25^, containing N- and C-terminal nuclear localization signals (NLS) with 3× FLAG repeats at the N-terminal part, was designed using a codon usage table derived from available gene models of *P. tricornutum* in GenBank, NCBI. The *diaCas9* gene was placed under the control of the *P. tricornutum LHCF2* promoter and has a *LHCF1* terminator sequence (Z24761.1). The whole module, promoter, *diaCas9* and terminator, was synthesized by GeneArt^®^ Services Thermo Fisher Scientific Inc. and Sac1 and Pst1 sites were included at 5′ and 3′ end of the module respectively, to facilitate cloning in a pBlueScript KS+ vector (Stratagene) where the BsaI restriction site in the ampicillin gene had been removed.

The *P. tricornutum* U6 snRNA promoter, located on Chr8 (complement pos. 239707–239986) acc: CM000611.1, was used for sgRNA expression. The RNA pol III transcription initiation site was estimated based on partially conserved 5′ DNA sequence motifs in the U6 gene, high-throughput DNA sequencing data, and a conserved guanine located 27 bp downstream of the TATA box. To enable easy generation of sgRNAs with customized target sites, the vector contains two BsaI restriction sites located at the 5′-end of the guide RNA, which allow ligation of adapters (target sites) with 5′ TCGA and AAAC overhangs^26^. The adapter for targeting the *CpSRP54* gene was made by annealing complementary oligos (1 μg of each oligo) with the above described overhangs (Supplementary Table 3) in an annealing reaction containing 1 × T4 Ligase Buffer (NEB) in a total volume of 50 μl. The annealing reaction was incubated in a heating block for 10 min at 85 °C, followed by slow cooling to room temperature (approx. 60 min). The resulting adapter was ligated into the pKS diaCas9_sgRNA plasmid using a molar vector to insert ratio of 1:20, and a T4 DNA ligase (Fermentas). A poly-T termination signal and 3′ sequences from the U6 gene was added at the 3′ end of the sgRNA module Chr8 (complement pos. 239266–239605). The module containing U6 snRNA promoter, sgRNA and U6 terminator was synthesized by Eurofins Genomic Services Ltd, with Pst1 and HindIII sites at 5′ and 3′ respectively to facilitate cloning. See Supplementary Fig. 2 for full DNA sequence of diaCas9 and sgRNA module. The pKS diaCas9_sgRNA plasmid vector will be made available through AddGene (Addgene ID: 74923).

### PAM-target site selection

A Perl-based script and a local Blast server (Ncbi-blast 2.2.27) were used to identify target sites with low homology to other genomic loci. PAM-target sites with high homology to other loci identified through Blast searches (blastN-short) were excluded. Due to the limitations of blastN-short to find sequences with mismatches, using a seed sequence of 12 to 14 bp (proximal to the PAM site), an additional string-based search was performed that screen for single bp mismatches located in the proximal seed region. Based on the string searches the PAM sites were rated according to their “uniqueness”.

### Biolistic transformation

*P. tricornutum* cell culture (500 μl) adjusted to 1 × 10^8^ cells/ml was plated on ½ f/2-Si, 1% agar plates and grown for 1 day. The pKS diaCas9_sgRNA vector was introduced to the cells by biolistic bombardment using the Biolistic PDS-1000/He Particle Delivery System (Bio-Rad, Hercules, CA, USA) as previously described^10^. The cells were co-transfected with the pAF6 vector^10^ to enable selection of transformants on ½ f/2-Si, 1% (w/v) agar plates containing 100 μg/ml zeocin (InvivoGen). Tungsten M17 microcarriers (Bio-Rad) were coated with 2.5 μg of each vector following the manufacturer’s instructions (Bio-Rad). Cells were transferred to selection plates 1 day after bombardment. Resistant colonies appearing 3–4 weeks after bombardment were transferred to liquid f/2 containing 50 μg/ml zeocin (InvivoGen).

### Screening for gene targeted mutations

Transformants were screened for DNA sequence mutations in the target region of the *CpSRP54* gene (Supplementary Fig. 3) by performing high-resolution melting (HRM) based PCR assays on the region spanning the target site of the Cas9-sgRNA complex. A small amount of cells derived from each transformant were lysed^7^. An amplicon with a size of approx. 500 bp surrounding the target site were amplified by PCR using the lysate as template and ExTaq DNA polymerase and Buffer system (TaKaRa). The PCR products were purified using Wizard^®^ SV Gel and PCR Clean-Up kit (Promega). The concentration of the purified PCR products were adjusted to 5 ng/μl, diluted 1:10^6^ and used as DNA template in following HRM analyses. An amplicon of approx. 100 bp surrounding the target site was amplified using the LightCycler 480 High Resolution Melting Master kit (Roche) following the manufacturer’s instructions. The PCR reactions and HRM curve analyses were performed on a LightCycler 96 instrument (Roche). Mutations were confirmed by Sanger sequencing. The 500 bp PCR product was cloned into pCR™4-TOPO^®^ TA vector (Invitrogen), and re-sequenced to get an overview of the different indels present within a colony. Primers used are presented in Supplementary Table 3.

### High blue light experiment

Six biological replicates of wild type (WT) cells and three biological replicates of each of the four different CpSRP54 mutant lines (M1, M4, M5 and M8) were subjected to 1 h of high intensity blue light (BHL; 500 μmol photon m^−2^ s^−1^) provided by a SL3500 LED light source (Photon System Instruments) at 15 °C. The PSII efficiency (maximum quantum yield; F_V_/F_M_) was measured at the end of a 3 min dark acclimation period using an AquaPen-C (Photon System Instruments) after 5 min, 0.5 h and 1 h of BHL exposure. After 1 h of BHL, cell cultures were returned to the previous growth conditions at 65 μmol photon m^−2^ s^−1^ white light (WL) at 22 °C. The PSII efficiency was measured 0.5 h, 1 h and 3 h into the recovery period.

### Isolation of “clean” CpSRP54 mutant lines

Highly diluted cell suspensions (~200 cells/ml; 500 μl) from five (M1, M2, M4, M5, M8) of the eight original mutant cultures were plated on ½ f/2-Si, 1% agar plates containing 100 μg/ml zeocin (InvivoGen). Clean CpSRP54 mutant lines with biallelic mutations originating from single cells, were isolated and identified by repeating the screening process described above on the resulting colonies.

### RNA isolation and quantitative real-time PCR

Harvesting of three biological replicates of WT and clean CpSRP54 mutant cultures (M1.1, M2.1, M4.1, M5.1, M8.1) grown at 22 °C, 65 μmol photon m^−2^ s^−1^, and subsequent RNA isolation, quantification and verification of RNA integrity were performed as described in Nymark *et al*.^24^. Reverse transcription of 1 μg of total RNA from all samples was performed with QuantiTect Reverse Transcription kit (Qiagen) following the recommended protocol. Reactions where the reverse transcriptase had been omitted were included for all samples to be used as genomic DNA controls during the quantitative real-time PCR (qRT-PCR) analyses. The qRT-PCR reactions were performed on a LightCycler 96 instrument (Roche) as described in Nymark *et al*.^24^, using cDNA diluted 1:10 as template. Forward and reverse primers used are listed in Supplementary Table 3. Quantification cycle (Cq) – values were determined using the LightCycler^®^ 96 Software.

### Cell divisions per day

Algal cells were counted by a NovoCyte^TM^ flow cytometer (ACEA Biosciences) to calculate the cell division per day in three biological replicates of WT and clean CpSRP54 mutant cultures (M1.1, M2.1, M4.1, M5.1, M8.1) grown under dim WL conditions (16 h photoperiod, 22 °C, 65 μmol photon m^−2^ s^−1^). Samples were excited by a 488 nm laser and chlorophyll fluorescence emission collected on a detector with a 675/30 nm bandpass filter.

## Additional Information

**How to cite this article**: Nymark, M. *et al*. A CRISPR/Cas9 system adapted for gene editing in marine algae. *Sci. Rep.* **6**, 24951; doi: 10.1038/srep24951 (2016).

## Supplementary Material

Supplementary Information

## Figures and Tables

**Figure 1:**
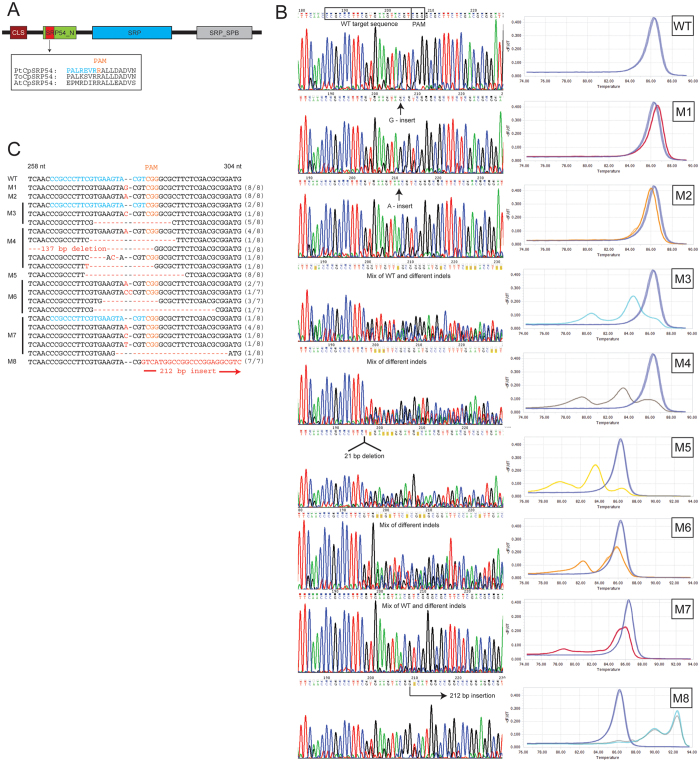
Genome editing and screening of *CpSRP54* gene mutants. (**A**) Schematic presentation of the CpSRP54 protein. The area of the protein corresponding to the target region for CRISPR/Cas9 based gene editing is located within the conserved SRP54_N domain (red highlighting). CLS: Chloroplast localization signal; SRP54_N; SRP54 N-terminal helical bundle domain; SRP: SRP GTPase containing domain; SRP_SPB: SRP54 signal peptide binding domain (**B**) Left side: Sequence chromatograms produced by direct sequencing of PCR product from WT cells and colonies containing cells with targeted mutations. Chromatograms produced by Chromas Lite version 2.0 software. Right side: High resolution melting analyses (HRM) data presented as normalized melting peaks generated from the corresponding PCR product. WT profiles (purple lines) are included in each plot to visualize the differences in melting behaviour between WT amplification products and products containing indels. (**C**) Overview of indels generated by transformation of *P. tricornutum* with the pKS diaCas9_sgRNA construct targeting the *CpSRP54* gene. Sequences represent cloned PCR products from colonies exhibiting targeted mutations. The number of times identical sequences were detected within the same colony is indicated in brackets to the right. Colour coding: Blue: WT target sequence; Orange: PAM sequence; Red dashes: Deleted bases; Red letters: Inserted bases.

**Figure 2:**
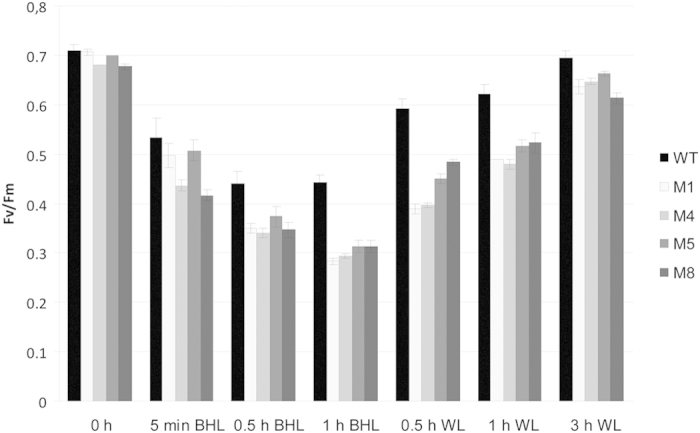
Photosynthetic efficiency after high intensity blue light exposure and recovery in dim white light. The photosynthetic efficiency (F_v_/F_m_) was determined in wild type cultures (WT; n = 6) and cultures containing CpSRP54 CRISPR/Cas9 mutants (M1, M4, M5 and M8; n = 3) after a 3 min dark acclimation period. Prior to the measurements the cells were exposed to blue high light (BHL; 500 μmol m^−2^ s^−1^) for 5 min, 0.5 h and 1 h with a following recovery in dim white light (WL; 65 μmol m^−2^ s^−1^) for 0.5 h, 1 h and 3 h. Values are presented with ±SD bars.
