# Supplementary material for: A CRISPR/Cas9 system adapted for gene editing in marine algae
Source: Sci Rep. 2016 Apr 25;6:24951. doi: 10.1038/srep24951 (PMC4842962; doi:10.1038/srep24951)
Supplement: Supplementary Information [file srep24951-s1.pdf]

Supplementary information.

A CRISPR/Cas9 system adapted for gene editing in marine algae.

Marianne Nymark, Amit Kumar Sharma, Torfinn Sparstad, Atle M. Bones and Per Winge.

Department of Biology, Norwegian University of Science and Technology, N-7491 Trondheim, Norway.

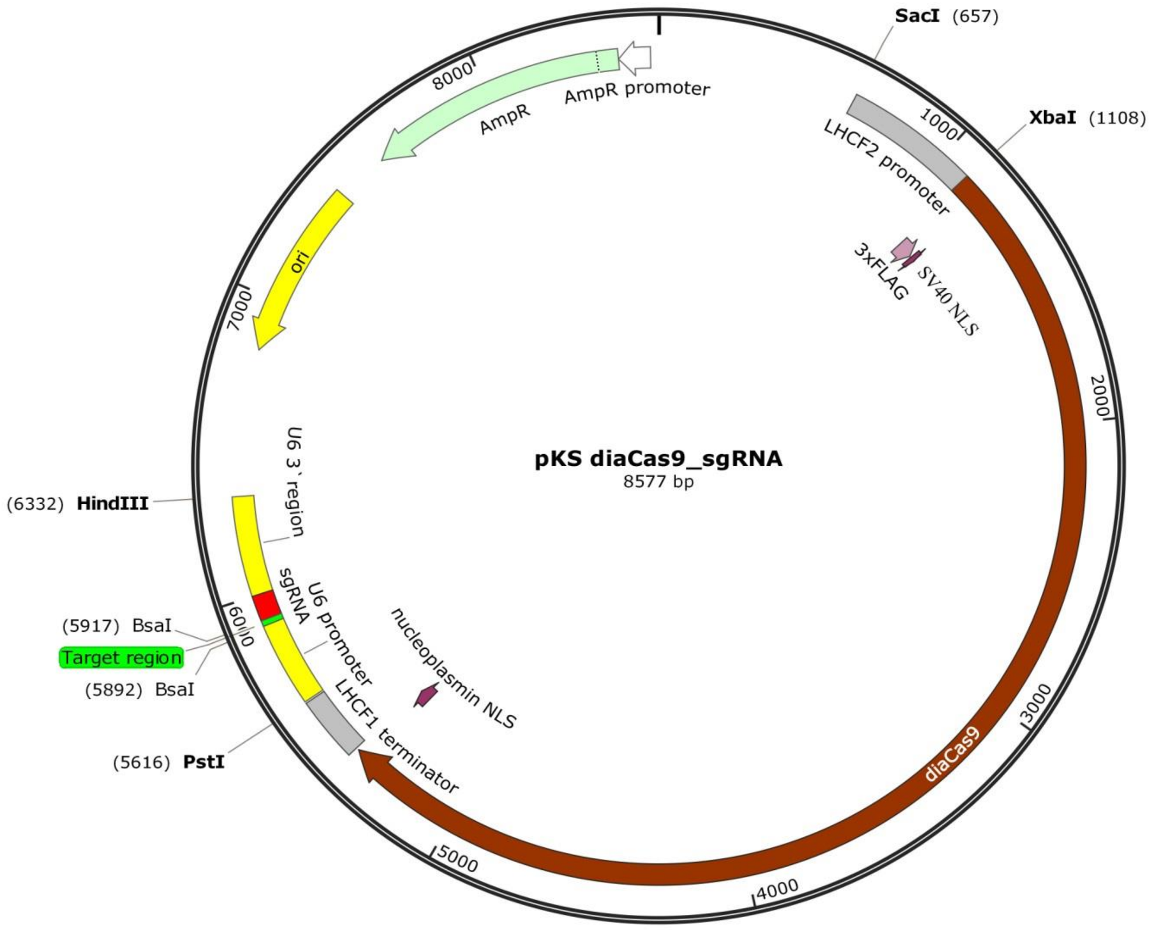

Supplementary Figure 1. Map of the pKS diaCas9_sgRNA plasmid.

>LHCF2 promoter-diaCas9-LHCF1 terminator

GAGCTCaatctcgcctattcatggtgtataaaagttcaacatccaaagctagaacttttg
gaaagagaaagaatatccgaatagggcacggcgtgccgtattgttggagtggactagcag
aaagtgaggaaggcacaggatgagttttctcgagacacatagcttcagcgtcgtgtaggc
taggcagaggtgagttttctcgagacataccttcagcgtcgtcttcactgtcacagtcaa
ctgacagtaatcgttgatccggagagattcaaaattcaatctgtttggacctggataaga
cacaagagcgacatcctgacatgaacgccgtaaacagcaaatcctggttgaacacgtatc
cttttggggggcctccgctacgacgctcgctccagctggggcttccttactatacacagcg
cgcatatttcacggttgccagaagtcaagATGGCC**TCTAGA**ATGGACTATAAGGACCACG
ACGGAGATTACAAGGATCATGATATTGATTACAAAGACGATGATGATAAAATGGCCCCAA
AGAAGAAGCGGAAAGTCGGTATACACGGAGTCCCAGCAGCCGACAAGAAGTACTCGATTG
GATTGGACATCGGCACCAACTCTGTTGGATGGGCCGTCATCACGGACGAGTACAAGGTGC
CCAGCAAGAAATTCAAAGTACTGGGAAACACTGACCGCCATTCGATCAAGAAGAATTTGA
TTGGAGCTTTGCTGTTTGACAGCGGCGAAACAGCGGAAGCCACGCGGCTTAAACGAACCG
CGAGACGAAGATATACGCGACGGAAGAACCGTATCTGCTATCTACAAGAGATTTTCTCGA
ATGAGATGGCAAAAGTCGATGACTCTTTCTTTCACCGATTGGAAGAATCCTTCCTCGTTG
AAGAGGATAAGAAACATGAGCGGCACCCGATATTTGGAAACATTGTGGACGAGGTGGCTT
ACCATGAGAAATACCCGACTATTTACCACCTGCGAAAGAAATTGGTGGACTCGACGGACA
AAGCGGATCTTCGCCTAATCTATTTGGCGCTAGCACATATGATTAAGTTTCGCGGCCACT
TCTTGATCGAAGGCGACTTGAATCCGGACAACAGTGATGTTGACAAACTGTTTATTCAAC
TTGTACAAACTTACAACCAGCTCTTTGAAGAAAATCCCATTAACGCTTCGGGAGTGGACG
CGAAAGCCATCCTTTCTGCCCGACTCAGCAAGAGCCGACGCCTAGAAAATTTGATTGCGC
AACTTCCCGGCGAGAAAAAGAATGGACTTTTTGGAAATCTTATTGCTTTGTCGCTGGGCC
TTACGCCGAACTTTAAGAGCAACTTCGACCTTGCGGAAGATGCCAAATTGCAACTGAGTA
AAGACACATATGACGACGACCTCGACAATCTTTTGGCACAAATAGGAGATCAGTACGCCG
ACCTCTTTTTGGCGGCTAAGAACTTGTCCGACGCCATTCTGTTGTCGGACATCCTTCGAG
TTAATACGGAGATTACTAAGGCGCCCTTGAGCGCCTCTATGATCAAAAGATACGACGAGC
ATCACCAAGACCTGACCCTCCTTAAAGCTCTCGTCCGGCAACAGCTGCCTGAGAAGTACA
AAGAGATTTTCTTTGATCAAAGCAAAAACGGATACGCGGGCTACATTGACGGGGGAGCCT
CGCAAGAAGAGTTCTACAAGTTTATTAAACCGATCCTGGAAAAGATGGACGGCACGGAGG
AATTACTCGTGAAACTTAACCGAGAGGACCTTTTGCGTAAGCAACGGACTTTCGACAACG
GATCTATTCCGCACCAAATCCATCTCGGAGAATTGCACGCAATTCTGCGCCGGCAAGAAG
ATTTTTATCCATTTCTCAAGGACAACCGGGAAAAGATTGAGAAAATCTTAACCTTCCGCA
TACCCTACTACGTCGGCCCTTTGGCCAGGGGAAATTCGAGATTTGCCTGGATGACGCGAA
AGAGTGAGGAAACTATTACTCCCTGGAATTTCGAAGAAGTGGTAGACAAAGGAGCTTCCG
CGCAATCCTTTATCGAGCGCATGACCAACTTCGATAAGAACCTCCCGAATGAGAAAGTCC
TACCCAAGCATTCGTTGCTCTACGAATACTTTACGGTGTATAATGAGCTGACTAAAGTCA
AATACGTTACAGAGGGAATGCGAAAACCGGCTTTCCTCTCGGGAGAGCAGAAAAAGGCCA
TAGTCGATCTTCTCTTTAAAACGAACCGTAAAGTTACCGTGAAACAACTAAAAGAGGACT
ACTTCAAGAAAATCGAATGCTTTGACTCCGTTGAAATTTCCGGCGTTGAAGATCGGTTCA
ATGCCTCCTTGGGAACATACCACGATCTCCTGAAAATTATCAAAGACAAGGACTTTCTTG
ACAATGAGGAAAACGAGGACATTCTTGAAGATATTGTCTTGACGCTAACACTTTTTGAGG
ATCGAGAGATGATTGAGGAACGCCTTAAAACCTATGCGCATCTGTTCGACGACAAAGTTA
TGAAGCAATTGAAGCGCCGGAGATATACGGGATGGGGAAGGTTGAGTCGGAAACTGATAA
ATGGCATACGCGATAAGCAGTCCGGCAAAACAATTCTCGATTTCTTGAAGTCCGACGGAT
TTGCGAATCGAAACTTCATGCAACTGATCCATGACGATTCGTTGACCTTTAAAGAGGACA
TTCAAAAAGCCCAAGTATCCGGACAAGGAGATTCTTTGCACGAGCATATTGCGAATCTTG

CTGGAAGTCCGGCTATTAAAAAGGGCATTCTCCAGACAGTAAAGGTCGTGGATGAACTCG
TTAAAGTTATGGGCCGACACAAACCCGAGAACATAGTCATCGAAATGGCGAGAGAGAACC
AGACCACCCAAAAGGGACAGAAAAATTCACGCGAGCGAATGAAGCGGATTGAAGAGGGGA
TCAAAGAGCTTGGCAGCCAAATTCTGAAAGAACACCCGGTTGAAAACACCCAATTGCAGA
ACGAGAAGCTCTACCTGTACTATCTTCAAAATGGGCGTGATATGTACGTCGATCAAGAAT
TGGACATCAACCGGCTATCCGATTACGATGTGGACCATATTGTTCCTCAGTCGTTTCTCA
AAGACGACTCCATAGACAATAAGGTTCTAACGAGAAGCGACAAGAACCGCGGCAAATCAG
ACAACGTCCCCTCCGAGGAAGTAGTTAAGAAAATGAAGAATTACTGGCGCCAATTGCTGA
ACGCTAAGCTCATTACCCAAAGAAAGTTTGACAATTTGACGAAAGCCGAGCGAGGCGGGC
TCTCGGAACTGGATAAGGCCGGCTTCATTAAAAGACAATTGGTGGAAACGCGACAAATCA
CAAAGCACGTCGCACAGATTCTCGACTCCCGCATGAACACTAAGTATGACGAGAATGACA
AACTAATCCGGGAAGTCAAAGTGATTACCTTGAAGTCCAAGCTGGTTTCCGATTTTCGTA
AAGATTTTCAGTTTTACAAAGTTCGCGAGATCAACAACTACCATCACGCGCACGACGCTT
ACTTGAACGCTGTCGTGGGAACGGCCTTGATTAAAAAATACCCTAAGCTGGAAAGCGAGT
TCGTTTACGGCGATTACAAGGTGTATGACGTACGCAAAATGATCGCGAAGTCAGAGCAGG
AAATTGGCAAAGCTACTGCGAAGTACTTCTTTTATAGCAACATAATGAATTTTTTCAAAA
CGGAGATTACCCTCGCTAACGGCGAGATTCGCAAGCGCCCTCTTATCGAGACAAACGGCG
AAACGGGGGAGATTGTCTGGGATAAAGGCCGCGATTTTGCGACTGTGCGGAAAGTCTTGT
CGATGCCGCAAGTGAATATAGTCAAAAAGACGGAGGTGCAAACAGGCGGCTTTTCGAAAG
AATCTATCCTTCCCAAAAGGAACTCTGATAAGTTGATTGCGCGAAAAAAGGATTGGGACC
CTAAGAAGTATGGCGGCTTTGACAGCCCGACCGTAGCATATTCTGTCCTTGTGGTGGCCA
AAGTCGAAAAGGGCAAATCCAAGAAACTGAAGAGTGTGAAAGAATTGCTTGGGATTACGA
TCATGGAAAGATCATCTTTTGAGAAGAATCCCATCGACTTTCTCGAAGCTAAAGGCTACA
AAGAAGTGAAAAAGGACCTGATTATCAAGTTGCCTAAGTACTCCTTGTTTGAGCTGGAAA
ATGGCCGTAAGCGAATGCTCGCTTCTGCCGGCGAACTCCAGAAGGGAAACGAATTGGCGC
TGCCCTCCAAATATGTTAACTTTCTTTACCTTGCCTCGCACTATGAGAAGCTCAAGGGCT
CCCCCGAGGATAATGAGCAAAAACAACTGTTTGTAGAACAGCATAAGCACTACTTGGACG
AGATCATTGAGCAAATTAGCGAGTTCTCCAAGCGAGTCATCCTTGCCGACGCTAATCTGG
ACAAAGTCCTGTCCGCCTACAATAAGCACCGCGATAAGCCAATCCGAGAGCAAGCGGAGA
ATATTATCCACTTGTTTACGCTCACCAATCTTGGAGCCCCTGCCGCCTTTAAGTACTTTG
ACACCACCATCGACCGCAAGAGGTACACCAGCACCAAAGAAGTCTTGGACGCCACCCTCA
TTCACCAATCAATCACCGGCTTGTACGAGACACGCATCGACCTGTCTCAACTCGGAGGCG
ACAAAAGGCCGGCGGCCACGAAAAAGGCCGGCCAGGCAAAAAAGAAAAAGTAAtct**GGAT**
CCctacctcgactttggctgggacactttcagtgaggacaagaagctccagaagcgtgct
atcgaactcaaccagggacgtgcggcacaaatgggcatccttgctctcatggtgcacgaa
cagttgggagtctctatccttccttaaaatttaattttcattagttgcagtcactccgc
tttggtttcacagtcaggaataacactagctcgtcttca**CTGCAG**

LHCF2 promoter and LHCF1 terminator sequences: lower case letters; diaCas9 sequence: upper case letters; restriction enzyme recognition sites: bold upper case letters.

>U6 promoter-sgRNA-U6 3′ region

CTGCAGgttggctcggaagttggtgttgacggtgagctggaaattggttgtcggtcactg
ctagcgagaagaaaacggaggacagaaggaagtgaaactcggttcgttctcgacagcctc
actgtcaatatgctcattttcaatccttagcgcttttaatgtcgaattgacggtaaattg
aataggatctataatatctacaaggtactttgacacgccaagtattcattgttagtcaac
aatatttagagctttataaggtcaaaaaacaccttcaaagtcgag**GAGACC**GAGAGAG**G**
GTCTCAGTTTTAGAGCTAGAAATAGCAAGTTAAAATAAGGCTAGTCCGTTATCAACTTGA
AAAAGTGGCACCGAGTCGGTGCttttttttagaaccgctcacccatgctatcgtatgtca
tttacattgacaaactgctatgaatgcgtttgcgataacttcagctcgctgaagccctag
acatttcgtttgactaaattgttttccggttgctttactaccgaccggtctgcgggtagc
atgtcttggcttttgcgagttcgagagaatttatggacaagtgcgtttacagtttggaac
acgagtgtgagtgagcggaattggctatctataacattcttatttcaggaggctggtgat
tagcagaattttcaaagatctcgaatctaaaatcgccatgggctcgacgaacttttctgc
AAGCTT

U6 promoter and U6 3’ region: in lower case letters; sgRNA sequence: upper case letters; restriction enzyme recognition sites: bold upper case letters.

Supplementary Figure 2. DNA sequence of diaCas9 and sgRNA module.

>CpSRP54 coding sequence [Phaeodactylum tricornutum CCAP 1055]

ATGAGACTCCAATCGGGATGTGTCTTGACGCTACTCGCCGCGACGTTCTATCCCTCTACA
CAAGCTTTTTCGATATTTTCGGTCGGCCCTTCGTTTGCTAGCTCGTCGTTCGCGAGTCGC
GAGASGAGCGACGTTTCCCGGAAGTCCTACCGTAGCACTGGTAGTAATCTGAACATGATG
TTCGATCAACTCTCTGCCGCCATTTCGGATGTCGCAAAGAATCTCGGCCC*GAAACGACGT*
*ATGTCGGAGGC*TTCCATTCAACCCGCCCTTCGTGAAGTACGT**CGG**GCGCTTCT*CGACGCG*
*GATGTCAACGTTGA*CGTCGCTGATACGTTGATCGAAGGAGTACGTGCGCGAAGCCTCGGG
CAGGAAGTATTGGAAGGTGTTACCGCCGAACAACAATTTGTTAAAGCCATGTACGATGAG
TTGCTCGACATGATGGGAGGCGATTCGTCAGTACCGATGAGTGACGGGCCCAGCAATGTT
CCCGTTGCGACCTTGGCGTCTGGTACCGCAGCCGATCCCGCYGTCATCTTGCTTGCAGGT
TTGCAAGGTGCCGGTAAAACAACCGCAGCCGGGAAACTCGCTCTTTTTTTGAAAGAACGT
GAGGTCGATTACGATCAAGTTGCTGCTATGGGAGATGAAGTTAAAAATACACTGGCCAGT
CGGCTGCCTAGGCGAGAACGCAAGGTGTTGCTGGTAGCAGCAGATATATATCGGCCGGCT
GCTATCAAACAGCTGCAAGTACTCGGAGAGAGCATTGGGGTAGAGGTATTTACTAAAGGA
ACGGATGTTGATCCGGTGGAAATTGTCAACGCAGGAATTCAAAAAGCTCGAGATGAAGGA
TATGATACCGTGATTGTGGATACAGCGGGTCGTCAAGTTATCGACACGGATCTCATGGAC
GAACTCCAGCGCATGAAAAGAGCTGCTAGTCCACAAGAAACTTTGTTGATCGTTGACGCC
ATGACCGGTCAGGAAGCTGCATCCTTGACGGCCGCTTTTGATTCCGCCATCGGACTAACA
GGTGCAATTTTAACTAAAATGGATGGCGATTCTCGTGGCGGTGCCGCTGTGAGCGTCCGA
GGGGTAAGTGGTAAGCCTATTAAATTCGTTGGAACCGGTGAAAAAACAGCCGATCTGGAA
CCGTTCTATCCGGATCGTATGGCAAGTCGCATTCTCGGTATGGGAGATGTAGTAAGCTTA
GTTGAAAAAGCCGCATCAGAGGTTTCAGATGCTGACGCACTCAAGATGCAGCAAAAAATG
CTTGACGCCAGCTTCGATTTCGATGACTTCGTGAAACAATCCGAGCTTGTCACCAAAATG
GGAAGCGTTGCCGGAATCGCGAAACTAATGCCTGGTATGGCCAATCAGCTCAACATGAAT
CAGATTCGTGAAGTCGAAGCTCGATTAAAGAAAAGCAAATCCATGATCTCAAGTATGACC
AAAAAGGAACGGGCAAACCCCGAGCTTTTGATCAAGGATTCTAGCGCCCGTTCTCGCTTA
ATTCGAATTACAAAAGGATCGGGCTGTGGGCTGGATGAAGGGCAACAGTTTATGAGCGAG
TTTCAGCGTATGAAAACCATGATGAGTCGAATGCAAAAGCAAATGGGCGGGAAGATGGAC
CCTTCCGCTGCCATGGGACCGGGCACCGAGGCAGAAATGCCCGACATGGGCAATCGAGCA
ATGCGACGAGCGGCGAAGAAATCAAAGAACAAGGCACGAGGCGGTTTTGGCGGTTTTGGC
TAA

Supplementary Figure 3. Coding sequence of *P. tricornutum* CpSRP54.

Supplementary Table 1. Quantification cycle (Cq) – values indicating gene expression of Cas9, sgRNA and LHCX1 genes. SD=standard deviation.

	Cq Cas9	SD Cas9	Cq sgRNA	SD sgRNA	Cq LHCX1	SD LHCX1
WT	-	-	-	-	16.3	0.33
CpSRP54 M1.1	19.4	0.22	14.9	0.16	17.2	0.69
CpSRP54 M2.1	16.6	0.64	17.4	0.36	17.2	1.23
CpSRP54 M4.1	19.3	0.19	16.1	0.19	17.5	0.36
CpSRP54 M5.1	17.6	0.21	14.6	0.28	15.8	0.19
CpSRP54 M8.1	-	-	18.5	0.09	17.6	0.32

Supplementary Table 2. Cell divisions per day in WT and clean CpSRP54 mutant lines.

	Divisions day^{-1}	SD (n=3)
WT	2.0	0.07
CpSRP54 M1.1	1.5	0.09
CpSRP54 M2.1	1.8	0.12
CpSRP54 M4.1	1.6	0.08
CpSRP54 M5.1	2.0	0.02
CpSRP54 M8.1	1.5	0.03

Supplementary Table 3. Adapter sequences, PCR, HRM, qRT-PCR and sequencing primers.

Primer name	Orientation	Sequence	Amplicon size (bp)
Pt35185_PF1	forward	TCGACCGCCCTTCGTGAAGTACGT	adapter
Pt35185_PR1	reverse	AAACACGTACTTCACGAAGGGCGG	adapter
Pt35185_G1F	forward	ACGTTCTATCCCTCTACACAAG	583
Pt35185_G1R	reverse	CAGCAACTTGATCGTAATCGAC	
HRM35185.1_F	forward	GAAACGACGTATGTCGGAGGC	84
HRM35185.1_R	reverse	TCAACGTTGACATCCGCGTCG	
qCasF2	forward	CTTGCGGAAGATGCCAAATTGC	54
qCasR1	reverse	GAGGTCGTCGTCATATGTGTCT	
Pt35185_PF1	forward	TCGACCGCCCTTCGTGAAGTACGT	78
qsgRNA _R	reverse	TCAAGTTGATAACGGACTAGCC	
M13 Rev (-29)		CAGGAAACAGCTATGACC	

Supplementary Figure 1. Map of the pKS diaCas9_sgRNA plasmid. diaCas9: diatom codon optimized Cas9 with *P. tricornutum* LHCF2 promoter and LHCF1 terminator; sgRNA: single guide RNA with *P. tricornutum* U6 promoter and U6 3’ region; 3xFLAG: three tandem FLAG® epitope tags; SV40 NLS: nuclear localization signal of SV40 large T antigen; nucleoplasmin NLS: bipartite nuclear localization signal from nucleoplasmin. The plasmid map was created with SnapGene software.

Supplementary Figure 2. DNA sequence of diaCas9 and sgRNA module.

Supplementary Figure 3. Coding sequence CpSRP54 (Phatr2_35185) from *P. tricornutum*, chromosome 7 (NC_011675.1). PAM target site in bold letters. Positions of primer sites used for PCR are underlined. Positions of primer sites used for HRM analyses are underlined and in italic.

Supplementary Table 1. Quantification cycle (Cq) – values indicating gene expression of Cas9, sgRNA and LHCX1 genes. The presented Cq-values for the Cas9, sgRNA and the endogenous *P. tricornutum* gene LHCX1, are the mean values of three biological replicates of WT and clean CpSRP54 mutant cultures.

Supplementary Table 2. Cell divisions per day in WT and clean CpSRP54 mutant lines. The presented cell division values are the mean of three biological replicates of WT and clean CpSRP54 mutant cultures grown under dim WL conditions (16 h photoperiod, 22°C, 65 µmol photon m^{-2} s^{-1}).

Supplementary Table 3. Adapter sequences, PCR, HRM, qRT-PCR and sequencing primers.
